# Supplementary material for: SARS-CoV-2 detection by a clinical diagnostic RT-LAMP assay
Source: Wellcome Open Res. 2021 May 21;6:9. Originally published 2021 Jan 21. [Version 2] doi: 10.12688/wellcomeopenres.16517.2 (PMC8170534; doi:10.12688/wellcomeopenres.16517.2)
Supplement: Peer review history

# Peer review: Reviewer response for version 2

**DOI:** 10.21956/wellcomeopenres.18513.r44001

**Published:** 2021-06-01

**Authors:** Viet Loan Dao Thi, Michael Knop

**Affiliations:** 1 Center of Infectious Diseases, Virology, Heidelberg University Hospital, Heidelberg, Germany; 2 Center for Molecular Biology of Heidelberg University (ZMBH), Heidelberg, Germany

We thank the authors for fully addressing our comments.

 Regarding the new chapter on the RNA extraction-free procedure, we would recommend citing and acknowledging the work of many other groups who have published similar protocols in the meantime.

Is the rationale for developing the new method (or application) clearly explained?

Yes

Is the description of the method technically sound?

Yes

Are the conclusions about the method and its performance adequately supported by the findings presented in the article?

Yes

If any results are presented, are all the source data underlying the results available to ensure full reproducibility?

Yes

Are sufficient details provided to allow replication of the method development and its use by others?

Yes

Reviewer Expertise:

Molecular virology and diagnostics.

We confirm that we have read this submission and believe that we have an appropriate level of expertise to confirm that it is of an acceptable scientific standard.

# Peer review: Reviewer response for version 2

**DOI:** 10.21956/wellcomeopenres.18513.r44002

**Published:** 2021-05-28

**Authors:** Leyi Wang, Vanessa Revindran-Stam

**Affiliations:** 1 Veterinary Diagnostic Laboratory, University of Illinois at Urbana-Champaign, Urbana, IL, USA

I am fine with the changes made by the authors. Thanks.

Is the rationale for developing the new method (or application) clearly explained?

Yes

Is the description of the method technically sound?

Yes

Are the conclusions about the method and its performance adequately supported by the findings presented in the article?

Yes

If any results are presented, are all the source data underlying the results available to ensure full reproducibility?

Yes

Are sufficient details provided to allow replication of the method development and its use by others?

Partly

Reviewer Expertise:

Molecular virology and clinical diagnostics.

We confirm that we have read this submission and believe that we have an appropriate level of expertise to confirm that it is of an acceptable scientific standard.

# Peer review: Reviewer response for version 1

**DOI:** 10.21956/wellcomeopenres.18193.r43084

**Published:** 2021-03-30

**Authors:** Leyi Wang, Vanessa Revindran-Stam

**Affiliations:** 1 Veterinary Diagnostic Laboratory, University of Illinois at Urbana-Champaign, Urbana, IL, USA

Till March 23, 2021, SARS-COV-2 global pandemic has caused infection of over 124 million and over 2.7 million deaths. It is critically important to mitigate transmission through detection and isolation. The current pandemic has made it widely important for fast, reliable and relatively cheap, alternative methods of detecting the SARS-CoV-2 infection. Numerous diagnostic methods for detection of virus genome and antigens have been developed. Molecular methods are most commonly used due to a higher level of sensitivity. The manuscript by Buck *et al*. reports evaluation of a clinical diagnostic RT-LAMP assay for detection of SARS-CoV-2. The authors performed sensitivity, specificity, and clinical validation. The authors used NEB LAMP reagent and one set of N gene primers and 18S primers reported from previous studies. They used 24 RNA samples with half positive for initial validation. Through testing water and RNA elution buffer, they optimized the assay with reduced reaction time to minimize background noise. They tested different species of human coronaviruses, and other human viruses and showed no cross reaction with non-SARS-CoV-2 viruses. They also determined the LOD of the assay and performed clinical validation on 37 and 71 samples tested by other labs. To address the false positive of RT-LAMP, evaluation of melting temperature was introduced in the procedure, however, there is no approach taken to address false negative due to the relative lower sensitivity compared with real-time qPCR.

**Major comments:**

1. A conventional real-time PCR enables multiplex capability, how can the validated assay of running two separate PCRs for one sample improve the workflow? Based on the data (Figure 5A and 5B), it seems switching from real-time PCR to LAMP could potentially lose sensitivity, as RT-LAMP missed weak positives. In addition, the incubation time for 18S is shorter than that for SARS-CoV-2, which means not same procedure used and 18S may not serve well as an internal control. The abundance of 18S (Figure 1D) may not reflect the presence of any inhibition in the test. Please clarify.
2. As two sets of N gene primers in the reference 10 (Zhang *et al*.), this study uses one set of them, have the authors tested both of them? While the N gene is highly conserved among different coronaviruses, what is the sensitivity/reliability of using one set of primers? Other publications have noted the use of multiple primer sets to increase sensitivity of the RT-LAMP assay and especially with the increasing discovery of variants of SARS-CoV, it would be crucial to include a combination of target genes.
3. In the pages 3 and 9, the RT-LAMP assay procedure, 3 µL of the RNA sample is used, however, in page 8 of the manuscript file, and pages 3 and 5 of the Supplementary Method file, 4.5 µL of the RNA was used. It is kind of confused. What correct amount is used in the procedure?
4. In the Introduction section, they mentioned that the LAMP assay alleviates the need for a thermocycler qPCR system, as the processing time on machine is less 30 minutes. The sentence needs to be rewritten. Instead using a dry bath in the Reference 10, this study still uses thermocycler. qPCR using the new fast chemistry (like TaqCheck) on QuantStudio, only takes 43 minutes on PCR machine. Therefore, the developed procedure may not help much to alleviate the need for thermocyclers.
5. This study only has a very low number (37 and 71, a total of 108) of the samples used for clinical validation of RT-LAMP, compared with their large testing volume completed by RT-qPCR. This part needs including much more samples for the clinical validation.
6. On numerous occasions, the authors refer to the RNA extraction through the CCC pipeline. There is missing extraction method in the materials and methods section.

**Minor comments:**

1. In Page 3, the authors stated they provided the preliminary data about running RT-LAMP on samples without RNA extraction, where are these data?
2. In the page 16 of Supplementary Method file, result interpretation table did not include possibility of real SARS-CoV-2 positive, but Internal control negative. Please clarify it.
3. It is confused that Figure 1B has 4 positive and 4 negative, but Figure 1C-1E are 12 positive and 12 negative. Please include colorimetric readout result for all 12 samples. There are missing Ct values of these 12 positive samples determined by RT-qPCR.
4. Figure 2: The pink dotted line in 2A, B, D and E should be identified.
5. Figure 3, C was not labeled/marked for 18S graph. In addition, in the colorimetric run for the RT-LAMp 18S rRNA, the 5th well looks to be pink/red and not yellow, indicating that one sample did not give signal.
6. For colorimetric read-out results, inconsistent data format was presented as Figure 1B was tubes containing the whole reaction, however, Figure 3B and 3C, and Figure 4 B were bottom part of tube, plate, or others? These need clearly indication in the figure legend.
7. Figure 4, 1% Triton-X 100 was shown. It is unclear which step Triton-X 100 was used and what is the purpose of using it.

Is the rationale for developing the new method (or application) clearly explained?

Yes

Is the description of the method technically sound?

Yes

Are the conclusions about the method and its performance adequately supported by the findings presented in the article?

Yes

If any results are presented, are all the source data underlying the results available to ensure full reproducibility?

Yes

Are sufficient details provided to allow replication of the method development and its use by others?

Partly

Reviewer Expertise:

Molecular virology and clinical diagnostics.

We confirm that we have read this submission and believe that we have an appropriate level of expertise to confirm that it is of an acceptable scientific standard, however we have significant reservations, as outlined above.

# Response

**Published:** 2021-04-28

**Authors:** Enzo Poirier

**Affiliations:** The Francis Crick Institute, UK

*We thank all reviewers for their constructive comments and suggestions. We have addressed their points with additional data and revisions to the text, as detailed below. We hope that the reviewers will find the revised manuscript improved and acceptable for publication.*

 Major comments:

  

1. A conventional real-time PCR enables multiplex capability, how can the validated assay of running two separate PCRs for one sample improve the workflow? Based on the data (Figure 5A and 5B), it seems switching from real-time PCR to LAMP could potentially lose sensitivity, as RT-LAMP missed weak positives. In addition, the incubation time for 18S is shorter than that for SARS-CoV-2, which means not same procedure used and 18S may not serve well as an internal control. The abundance of 18S (Figure 1D) may not reflect the presence of any inhibition in the test. Please clarify.
*We have modified the manuscript to include further discussion of the advantages and disadvantages of the outlined RT-LAMP assay in comparison to conventional RT-qPCR.*
**
* Switching from RT-PCR to RT-LAMP would result in a loss in sensitivity. However, the loss in sensitivity is less than what is experienced for currently approved antigen tests and the assay provides other advantages. Supplies of RT-LAMP reagents, especially in the beginning of the pandemic, were more readily available, easier to acquire and markedly less expensive, than RT-PCR reagents due to less demand and markedly less expensive. The assay can also be performed without a thermocycler if need be.*
**
* In terms of the workflow, the assay can be run on either 96 or 384-well plate format with a thermocycler and both the SARS-CoV-2 and internal control reactions can be ran on the sample plate for the same amount of time as amplification results are only assessed at a defined data even if data is collected beyond that time point (e.g. N-gene assay, amplification before 25 minutes and 18S rRNA assay, amplification before 20 minutes).*
2. As two sets of N gene primers in the reference 10 (Zhang et al.), this study uses one set of them, have the authors tested both of them? While the N gene is highly conserved among different coronaviruses, what is the sensitivity/reliability of using one set of primers? Other publications have noted the use of multiple primer sets to increase sensitivity of the RT-LAMP assay and especially with the increasing discovery of variants of SARS-CoV, it would be crucial to include a combination of target genes.
*We did initial tests with different primer sets that were originally denoted in Zhang et al. and have included this data in Supplement Figure 1 with an explanation of our rationale for moving forward with the particular N gene primers that we used throughout the rest of our studies.*
**
* In regards to current questions of reliability in light of new variants, Reviewer #1 had similar concerns and we have now included analyses that compare the mutational rate in the specific sequences that these primers target (see Supplemental Figure 2).*
**
* As mentioned with Reviewer #1, we have now included discussion of various strategies, including the use of multiple primer pairs, to increase the sensitivity of the RT-LAMP assay in the revised manuscript.*
3. In the pages 3 and 9, the RT-LAMP assay procedure, 3 µL of the RNA sample is used, however, in page 8 of the manuscript file, and pages 3 and 5 of the Supplementary Method file, 4.5 µL of the RNA was used. It is kind of confused. What correct amount is used in the procedure?
*We apologise for the confusion: the standard RNA input amount is 4.5 µL, however some experiments were performed using 3 µL of input due to limitations in sample availability. Experiments that used 3 µL as opposed to the standard amount have been denoted in the corresponding figure legends and text.*
4. In the Introduction section, they mentioned that the LAMP assay alleviates the need for a thermocycler qPCR system, as the processing time on machine is less 30 minutes. The sentence needs to be rewritten. Instead using a dry bath in the Reference 10, this study still uses thermocycler. qPCR using the new fast chemistry (like TaqCheck) on QuantStudio, only takes 43 minutes on PCR machine. Therefore, the developed procedure may not help much to alleviate the need for thermocyclers.
*We have modified the specific sentence mentioned from the introduction and have expanded our discussion in the revised text of the advantages and disadvantages of RT-LAMP. There are still instances where access to a thermocycler may not be readily available and a procedure that can be carried out without one if necessary (RT-LAMP) would be a valued asset. We have mentioned this fact in our revised discussion.*
5. This study only has a very low number (37 and 71, a total of 108) of the samples used for clinical validation of RT-LAMP, compared with their large testing volume completed by RT-qPCR. This part needs including much more samples for the clinical validation.
*We acknowledge that increasing the number of clinical samples tested by RT-LAMP would allow more robust clinical validation of the assay’s performance. However, as seen in our response to Reviewer #1, point 7, the assay was ultimately not adopted for use at the institute. We have included discussion of this particular issue in the revised manuscript.*
6. On numerous occasions, the authors refer to the RNA extraction through the CCC pipeline. There is missing extraction method in the materials and methods section.
*We apologise for not including this information in the original submission and have amended the methods accordingly.*

Minor comments:

  

1. In Page 3, the authors stated they provided the preliminary data about running RT-LAMP on samples without RNA extraction, where are these data?
*We apologise that these data were not included in the original submission. They now appear in new Supplemental Figure 6.*
2. In the page 16 of Supplementary Method file, result interpretation table did not include possibility of real SARS-CoV-2 positive, but Internal control negative. Please clarify it.
*Please see our response to Reviewer #1, point 12, first paragraph.*
3. It is confused that Figure 1B has 4 positive and 4 negative, but Figure 1C-1E are 12 positive and 12 negative. Please include colorimetric readout result for all 12 samples. There are missing Ct values of these 12 positive samples determined by RT-qPCR.
*As the primary readout in Figure 1C-E was Syto9 amplification, we do not have the corresponding images demonstrating the colorimetric conversions.*
**
* The Ct values from Figures 1C and 1D are displayed in Figure 1E (grey coloured dots, refer to left y-axis) as indicated in the figure legend.*
4. Figure 2: The pink dotted line in 2A, B, D and E should be identified.
*We have removed the pink dotted line, which referred to the threshold set for the assay, from the indicated figures as its depiction is not necessary.*
5. Figure 3, C was not labeled/marked for 18S graph. In addition, in the colorimetric run for the RT-LAMp 18S rRNA, the 5th well looks to be pink/red and not yellow, indicating that one sample did not give signal.
*We thank the reviewer for their careful eye and have corrected the corresponding figure legend.*
**
* As for the 5 ^th^ well that appears lighter in hue compared to other samples, we have explained the discrepancy in our response to Reviewer #1 (see response 17, paragraph 3 and response 19)*
6. For colorimetric read-out results, inconsistent data format was presented as Figure 1B was tubes containing the whole reaction, however, Figure 3B and 3C, and Figure 4 B were bottom part of tube, plate, or others? These need clearly indication in the figure legend.
*We have amended the indicated figure legends as suggested to clarify how our colorimetric data are presented.*
7. Figure 4, 1% Triton-X 100 was shown. It is unclear which step Triton-X 100 was used and what is the purpose of using it.
*Please see our response to Reviewer #1, point 20.*

# Peer review: Reviewer response for version 1

**DOI:** 10.21956/wellcomeopenres.18193.r42423

**Published:** 2021-02-15

**Authors:** Viet Loan Dao Thi, Michael Knop

**Affiliations:** 1 Center for Molecular Biology of Heidelberg University (ZMBH), Heidelberg, Germany; 2 Center of Infectious Diseases, Virology, Heidelberg University Hospital, Heidelberg, Germany

Summary:

 In the ongoing pandemic, alternative methods to detect SARS-CoV-2 infections at a scalable, faster, and cheaper level, are still in need. In this manuscript, Buck *et al*. report on the use of the NEB warmstart enzyme preparation for colorimetric reverse transcription loop-mediated isothermal amplification (RT-LAMP) in combination with primers published by NEB (Zhang *et al.*) for the amplification and detection of SARS-CoV-2 viral RNA in clinical samples. For detection, a colorimetric implementation of the RT-LAMP assay (based on a pH shift and color change of an indicator dye upon amplification of nucleic acids) along with a fluorescent dye for DNA detection was used. The assay was tested for COVID-19 diagnostics and the report includes data on sensitivity, specificity as well as a positive sample control reaction. All established procedures were set up in agreement with the requirements for patient diagnostics, including SOPs for all relevant procedures. The authors furthermore claim that the cost of the assays would be a fraction of the ones used for RT-qPCR.

 Major points:

 The authors use colorimetric LAMP reagents but then add a green fluorescent dye for the detection of the fluorescence signal using a light cycler throughout their experiments, with the exception of the few figure panels where they show color images of the reaction tubes? Is this correct?

 Importantly, the authors advertise the speed of the RT-LAMP assay in the abstract (25 min). This is somewhat misleading to non-expert readers who might think that the total time of the procedure (from sample taking to result) takes only that long. But the time to isolate the RNA and to set up the assays also needs to be considered.

 The preprint in its current form appeared online on July 2020, and the authors claim clinical validation of the assay, higher speed, and almost similar sensitivity to qPCR at much lower costs. Given these advantages, may I ask whether the assay has been used for SARS-CoV-2 diagnostics? If yes, for how many samples? It would be essential to present such data including statistics from validation of LAMP positives by qPCR. On the other hand, if the assay was not used for diagnostics, it would be good to carefully explain the reasons behind it; otherwise, this may raise concerns about the validity of some of the data and acclaimed features of the assay.

 Average RT-qPCR assays using well-established primer sets meanwhile reach sensitivities where 10 molecules of the viral genome are detected in 95% of the cases, which for most RT-qPCR kits corresponds to a CT approx. = 37. This can be considered as standard. Is this given for the used PCR assay (CRICK BGI)?

 The manuscript does not elaborate on the used RNA isolation procedures. This should be included in the methods section. Different RNA extraction protocols exist, either using silica matrix columns or magnetic beads. These protocols allow purification and simultaneous concentration of nucleic acids relative to the original specimen. For diagnostic analysis of nucleic acids in SWAB specimen, a concentration factor of 2 to 5 usually applies. This yields a LOD of approx. 500 or fewer viral RNA molecules per ml of patient specimen for an RT-qPCR that uses 10 μl input volume. The authors here claim a sensitivity of the RT-LAMP of 11000-22000 molecules per ml patient specimen using a SARS-CoV-2 standard (Figure 4), while they claim a sensitivity up to Ct=35 with patient samples (Figure 5a). A Ct=35 in an RT-qPCR assay corresponds roughly to 40 molecules per 10 μl purified RNA, while only 3 μl are used for the RT-LAMP, which would imply a sensitivity limit of approx. 15 molecules per reaction. This is inconsistent with the data shown in Figure 4 and with previous reports for the N-gene LAMP primer sets used here, which was reported to have a sensitivity in the range of 100 molecules per reaction (e.g. ^1^). A closer inspection of the data shown in Figure 5a indicates that all RT-LAMP positive samples have CT<<32 (Figure 4a), but not enough samples in the CT range of 25 – 35 are shown to confidently estimate a CT value that corresponds to the LOD. These issues should be clarified and, if necessary, faulty standard calibrations or additional means to calibrate the sensitivity should be used.

 The authors represent a consortium that has generated more than 252000 RT-qPCR results since the beginning of the pandemic. Obviously, knowing the exact sensitivity limit of the RT-LAMP assay and the data of the RT-qPCR would permit an estimation of the false-negative rate of the RT-LAMP assay in a much more realistic scenario as compared to the limited number of samples presented here.

 The assay makes use of one primer set only for SARS-CoV-2 detection. Meanwhile, many more primer sets have been described, and a publication by NEB scientists ^2^ furthermore presented means to improve the sensitivity of the assay by adding GuHCL to the reaction buffer and by using multiple primer sets in combination. This could be discussed and the benefits of using better primers/conditions to be estimated.

 Specificity: The authors elude that the RT-LAMP assay does not cross-react with other viruses. If the authors really want to use this experiment as a valid assay to demonstrate the sensitivity of the RT-LAMP assay for SARS-CoV-2, they should provide evidence that the used samples indeed contain viral nucleic acids in sufficient numbers (> 10000 molecules per ml) of the patient specimen. Another issue that needs to be addressed is the relatively well-established propensity of RT-LAMP to produce false-positive results by spurious amplification (e.g. see ^3^). The frequency of these may be small, i.e. below 1%, and hence such cases escaped detection in this work. As shown by the authors, melting curves allow to identify those, but upscaling and the use of the colorimetric RT-LAMP will certainly bear the risk of false positives. Here, validation of positives by RT-qPCR could be used.

 Additional points:

 The authors mention twice preliminary data on an RNA-extraction free procedure. Where are these data? I suggest either adding the data or removing the corresponding sentences in the introduction and discussion.

 The number of cells in a swab can be affected by many factors. Even in the absence of human cells/detectable housekeeping genomes, viral RNA can be or should be present in a positive sample in the form of cell-free, viral particles. How do the authors suggest proceeding if 18S rRNA is not detected in a sample? How does the detection of the housekeeping gene affect the analysis of the clinical samples (e.g. in Figure 5)?

 For example, in Figure 3B. The fifth sample appears to be 18S rRNA negative, as the reaction is still pink. How is such a result taken into account?

 The citations are not exhaustive. Many manuscripts have been published meanwhile that make use of the colorimetric RT-LAMP assay for COVID-19 diagnostics. In addition, a description of the major differences or similarities to previously published work is missing.

 The manuscript would benefit from a report on the frequency of mutations in the binding sequences of the primer set used, which could lead to false negatives. This can easily be done using online resources ( https://www.gisaid.org or https://covidcg.org) and the great number of viral genomes sequenced in the UK.

 The authors need to report on measures to avoid contamination by LAMP products. This is an important point that may be less relevant for a diagnostic setting (since people are aware of this issue), but in an academic environment, researchers may need a reminder, which could be given for example by pointing out relevant literature ^4^ or by indicating strategies to eliminate those in the first place (UNG/UDG), or both.

 Specific comments to the Figures:

 Figure 1: Suggest moving primer sequences to a separate table.

 Figure 2: Panels D and E are redundant with Panels A and B as the threshold is indicated by the grey background (just add an explanation of the threshold to the legend). Panel C is also redundant and difficult to read, the information is already in A and B. Give an explanation of the false negatives on the 18S control, which already give fluorescent signals at 20 mins into the LAMP reaction. The primer design seems suboptimal as spurious amplification usually occurs at later time points. At what time point did the authors analyze the colorimetric read-out for 18S, e.g. in Fig 3B. Please define up to when is the reaction reliable.

 Suggest moving the melting curves from S1A to the main figures. When calculating sensitivity thresholds-does the melting curve change anything? Does it improve the sensitivity?

 Figure 3B, right panel: All different samples are just blue lines. Like this, it is impossible for the reader to understand which line corresponds to which sample. As the coloration is somewhat different between the samples as well as the time points where the fluorescent signal becomes positive, it would be good to see if some sort of correlation exists or not.

 Figure 4 C: I do not understand the reason behind treating already purified SARS-CoV-2 RNA with Triton X. This only makes sense in an extraction-free setting.

 Figure 4 D: When determining the RT-LAMP assay precision, why extracting the RNA 5 times independently and then running the assay? I expect higher variations from the RNA extraction than from the RT-LAMP assay itself, which should be highly stable and reproducible on purified samples unless the sample contains only very few amounts of target RNA and the detection can become stochastic.

Is the rationale for developing the new method (or application) clearly explained?

Yes

Is the description of the method technically sound?

Yes

Are the conclusions about the method and its performance adequately supported by the findings presented in the article?

Yes

If any results are presented, are all the source data underlying the results available to ensure full reproducibility?

Yes

Are sufficient details provided to allow replication of the method development and its use by others?

Yes

Reviewer Expertise:

Molecular virology and diagnostics.

We confirm that we have read this submission and believe that we have an appropriate level of expertise to confirm that it is of an acceptable scientific standard, however we have significant reservations, as outlined above.

## References

[1] : Rapid Molecular Detection of SARS-CoV-2 (COVID-19) Virus RNA Using Colorimetric LAMP. *medRxiv*.2020; 10.1101/2020.02.26.20028373 10.1101/2020.02.26.20028373

[2] : Enhancing Colorimetric LAMP Amplification Speed and Sensitivity with Guanidine Chloride. *bioRxiv*.2020; 10.1101/2020.06.03.132894 10.1101/2020.06.03.132894 32635743

[3] : High-surety isothermal amplification and detection of SARS-CoV-2, including with crude enzymes. *bioRxiv*.2020; 10.1101/2020.04.13.039941 10.1101/2020.04.13.039941

[4] : Amplicon contamination in labs masquerades as COVID19 in surveillance tests. *medRxiv*.2020; 10.1101/2020.12.08.20244525 10.1101/2020.12.08.20244525

# Response

**Published:** 2021-04-28

**Authors:** Enzo Poirier

**Affiliations:** The Francis Crick Institute, UK

*We thank all reviewers for their constructive comments and suggestions. We have addressed their points with additional data and revisions to the text, as detailed below. We hope that the reviewers will find the revised manuscript improved and acceptable for publication.*

 Major points:

  

1. The authors use colorimetric LAMP reagents but then add a green fluorescent dye for the detection of the fluorescence signal using a light cycler throughout their experiments, with the exception of the few figure panels where they show color images of the reaction tubes? Is this correct?
*Yes, as the reviewers have stated, we used colorimetric reagents combined with a green fluorescent DNA intercalating dye with amplification read-out by using a real-time PCR machine. Some figure panels feature the colorimetric conversion from these experiments. *
2. Importantly, the authors advertise the speed of the RT-LAMP assay in the abstract (25 min). This is somewhat misleading to non-expert readers who might think that the total time of the procedure (from sample taking to result) takes only that long. But the time to isolate the RNA and to set up the assays also needs to be considered.
*We thank the reviewer for their comment and have excluded the mention of the assay run time to help avoid confusion between the run-time of the RT-LAMP assay versus total procedure time from sample collection, RNA extraction, RT-LAMP, and results reporting. *
3. The preprint in its current form appeared online on July 2020, and the authors claim clinical validation of the assay, higher speed, and almost similar sensitivity to qPCR at much lower costs. Given these advantages, may I ask whether the assay has been used for SARS-CoV-2 diagnostics? If yes, for how many samples? It would be essential to present such data including statistics from validation of LAMP positives by qPCR. On the other hand, if the assay was not used for diagnostics, it would be good to carefully explain the reasons behind it; otherwise, this may raise concerns about the validity of some of the data and acclaimed features of the assay.
*We thank the reviewer for their suggestion and have modified the discussion to explain the reasons why the assay was not ultimately adopted at our institute.*
4. Average RT-qPCR assays using well-established primer sets meanwhile reach sensitivities where 10 molecules of the viral genome are detected in 95% of the cases, which for most RT-qPCR kits corresponds to a CT approx. = 37. This can be considered as standard. Is this given for the used PCR assay (CRICK BGI)?
*The LOD for the BGI’s Real-Time Fluorescent RT-PCR kit for detecting 2019-nCov (SARS-CoV-2) is 100 copies/mL, which is consistent with standard viral genome detection from other RT-qPCR kits (https://www.bgi.com/global/molecular-genetics/2019-ncov-detection-kit/).*
5. The manuscript does not elaborate on the used RNA isolation procedures. This should be included in the methods section. Different RNA extraction protocols exist, either using silica matrix columns or magnetic beads. These protocols allow purification and simultaneous concentration of nucleic acids relative to the original specimen.
*We apologise for not including the information in our original submission as the complete RNA isolation/purification procedure utilised by the institute’s RT-qPCR pipeline had already been published in its entirety. However, we have now included a brief description in the revised methods.*
6. For diagnostic analysis of nucleic acids in SWAB specimen, a concentration factor of 2 to 5 usually applies. This yields a LOD of approx. 500 or fewer viral RNA molecules per ml of patient specimen for an RT-qPCR that uses 10 μl input volume. The authors here claim a sensitivity of the RT-LAMP of 11000-22000 molecules per ml patient specimen using a SARS-CoV-2 standard (Figure 4), while they claim a sensitivity up to Ct=35 with patient samples (Figure 5a). A Ct=35 in an RT-qPCR assay corresponds roughly to 40 molecules per 10 μl purified RNA, while only 3 μl are used for the RT-LAMP, which would imply a sensitivity limit of approx. 15 molecules per reaction. This is inconsistent with the data shown in Figure 4 and with previous reports for the N-gene LAMP primer sets used here, which was reported to have a sensitivity in the range of 100 molecules per reaction (e.g. 1). A closer inspection of the data shown in Figure 5a indicates that all RT-LAMP positive samples have CT<<32 (Figure 4a), but not enough samples in the CT range of 25 – 35 are shown to confidently estimate a CT value that corresponds to the LOD. These issues should be clarified and, if necessary, faulty standard calibrations or additional means to calibrate the sensitivity should be used.
*We apologise for the confusion and have clarified our discussion of the assay’s LOD, including the acknowledgement that a true determination of the LOD/false negative rate would require additional clinical specimen testing. We thank the reviewer also for their careful analysis of Figure 5A. Samples that had Ct > 33, not 35 (a typo), were not consistently detected, which would be more consistent with the existing LOD determinations.*
**
* We have also modified the text to more explicitly state that the assay is less sensitive than RT-qPCR.*
7. The authors represent a consortium that has generated more than 252000 RT-qPCR results since the beginning of the pandemic. Obviously, knowing the exact sensitivity limit of the RT-LAMP assay and the data of the RT-qPCR would permit an estimation of the false-negative rate of the RT-LAMP assay in a much more realistic scenario as compared to the limited number of samples presented here.
*We acknowledge that knowing the exact sensitivity and running the RT-LAMP assay in parallel against RT-qPCR for the same samples would allow a true estimation of the false-negative rate of the RT-LAMP assay. However, as stated above in response 3, the assay was ultimately not adopted and therefore, we can only speculate. We have now included some discussion of this particular issue in the revised text.*
8. The assay makes use of one primer set only for SARS-CoV-2 detection. Meanwhile, many more primer sets have been described, and a publication by NEB scientists 2 furthermore presented means to improve the sensitivity of the assay by adding GuHCL to the reaction buffer and by using multiple primer sets in combination. This could be discussed and the benefits of using better primers/conditions to be estimated.
*In the first months of the pandemic during the initiation of our studies, we tested several LAMP primers specific for SARS-CoV-2 as outlined in Ref 1. We moved forward with our particular primers because they did not display aberrant non-specific amplification seen with the other primers. We have now mentioned this in the revised text and included these data  in new Supplement Figure 1.*
**
* As the reviewer has suggested, we have expanded our discussion of possible ways to optimise the assay.*
9. Specificity: The authors elude that the RT-LAMP assay does not cross-react with other viruses. If the authors really want to use this experiment as a valid assay to demonstrate the sensitivity of the RT-LAMP assay for SARS-CoV-2, they should provide evidence that the used samples indeed contain viral nucleic acids in sufficient numbers (> 10000 molecules per ml) of the patient specimen.
*We have included this information now in Figure 3B and Supplement Figure 4.*
10. Another issue that needs to be addressed is the relatively well-established propensity of RT-LAMP to produce false-positive results by spurious amplification (e.g. see 3). The frequency of these may be small, i.e. below 1%, and hence such cases escaped detection in this work. As shown by the authors, melting curves allow to identify those, but upscaling and the use of the colorimetric RT-LAMP will certainly bear the risk of false positives. Here, validation of positives by RT-qPCR could be used.
*We agree that samples suspected of being false-positives (e.g. late amplification) could be ruled out by validation with RT-qPCR and have included this suggestion in the text.*

Additional points:

  

1. The authors mention twice preliminary data on an RNA-extraction free procedure. Where are these data? I suggest either adding the data or removing the corresponding sentences in the introduction and discussion.
*We apologise for this oversight and have described the data now in the corresponding text for new Supplemental Figure 6.*
2. The number of cells in a swab can be affected by many factors. Even in the absence of human cells/detectable housekeeping genomes, viral RNA can be or should be present in a positive sample in the form of cell-free, viral particles. How do the authors suggest proceeding if 18S rRNA is not detected in a sample? How does the detection of the housekeeping gene affect the analysis of the clinical samples (e.g. in Figure 5)? For example, in Figure 3B. The fifth sample appears to be 18S rRNA negative, as the reaction is still pink. How is such a result taken into account?
*We acknowledge that SARS-CoV-2 could be present in samples in cell-free, viral particles that may not be associated with human cells. However, to avoid potential confusion during diagnosis between the following two scenarios: whether a patient’s sample may have been inadvertently contaminated during processing with SARS-CoV-2 versus detection of SARS-CoV-2 from a genuine nasopharyngeal swab, we explicitly state in the supplemental methods that a SARS-CoV-2 positive result would only be made if there was sufficient amplification in the internal control.*
**
* The analysis of the housekeeping gene should not affect the analysis of SARS-CoV-2 as they are carried out in separate reactions (excluding any possible errors associated with loading of the RNA input into the reaction).*
**
* The sample in Figure 3B was 18S rRNA positive and was one of the last samples to amplify (around 18 minutes) as seen in the corresponding amplification plot (now denoted by asterisk). One of the reasons we utilised a thermocycler for detection of Syto9 incorporation was to more accurately quantitate amplification during the assay because as the reviewer points out, sometimes a change in sample colour is not always obvious to the naked eye.*
3. The citations are not exhaustive. Many manuscripts have been published meanwhile that make use of the colorimetric RT-LAMP assay for COVID-19 diagnostics. In addition, a description of the major differences or similarities to previously published work is missing.
*We agree that many studies since our original submissions have been published and now reference a recent review that compares and contrasts various RT-LAMP assays for COVID-19 diagnostics that have been developed. We have revised our discussion to include a comparison of our assay against previous work.*
4. The manuscript would benefit from a report on the frequency of mutations in the binding sequences of the primer set used, which could lead to false negatives. This can easily be done using online resources (https://www.gisaid.org or https://covidcg.org) and the great number of viral genomes sequenced in the UK.
*We have undertaken an analysis of the frequency of mutations present in the binding sequences of the primers we utilised as the reviewers have suggested. These new data are included in Supplemental Figure 2 and the corresponding revised text.*
5. The authors need to report on measures to avoid contamination by LAMP products. This is an important point that may be less relevant for a diagnostic setting (since people are aware of this issue), but in an academic environment, researchers may need a reminder, which could be given for example by pointing out relevant literature 4 or by indicating strategies to eliminate those in the first place (UNG/UDG), or both.
*We have mentioned in the revised discussion the need and strategies to avoid contamination by LAMP products, including the relevant literature that the reviewer has brought our attention to.*

Specific comments to the Figures:

  

1. Figure 1: Suggest moving primer sequences to a separate table.
*For increased visibility, we have decided to continue highlighting the primers utilised in Figure 1.*
2. Figure 2: Panels D and E are redundant with Panels A and B as the threshold is indicated by the grey background (just add an explanation of the threshold to the legend). Panel C is also redundant and difficult to read, the information is already in A and B. Give an explanation of the false negatives on the 18S control, which already give fluorescent signals at 20 mins into the LAMP reaction. The primer design seems suboptimal as spurious amplification usually occurs at later time points. At what time point did the authors analyze the colorimetric read-out for 18S, e.g. in Fig 3B. Please define up to when is the reaction reliable.
*We have removed the grey background in Figures 2A and 2B and removed Figure 2C as suggested. Since Panels 2D and 2E were from separate experiments done in Figures 2A and 2B utilising the new detection threshold, we have chosen to keep them within the main figure.*
**
* We acknowledge that the 18S rRNA specific primers we adopted from a previous study were not exhaustively assessed in our own studies and may not be optimally designed, which may account for the exhibition of spurious amplification. However, the inclusion of the melting curve analyses should help distinguish these false positives from true positives.*
**
* The colorimetric reactions were visualised either after 25 minutes of amplification (N-gene SARS-CoV-2) or 20 minutes of amplification (18S rRNA). Although the images are included alongside the amplification plots (corresponding to Syto9 dye incorporation), positive amplification by Syto9 read-out was more sensitive than the subjective colour conversion read-out as explained in response 12.*
3. Suggest moving the melting curves from S1A to the main figures. When calculating sensitivity thresholds-does the melting curve change anything? Does it improve the sensitivity?
*We have done as the reviewer has suggested and modified Figure 2 to include the data featured in Figure S1A.*
**
* The inclusion of the melting curve analyses does not improve the sensitivity of the reaction (e.g. carrying out amplification for longer periods in the hopes of amplifying low copy number positives), but merely aids in distinguishing true amplification from false amplification. Samples that have copy numbers below the LOD do not appear to amplify with longer incubation times (data not shown).*
4. Figure 3B, right panel: All different samples are just blue lines. Like this, it is impossible for the reader to understand which line corresponds to which sample. As the coloration is somewhat different between the samples as well as the time points where the fluorescent signal becomes positive, it would be good to see if some sort of correlation exists or not.
*For the one sample that had late 18S rRNA amplification and concurrently, suboptimal colour change, we now denote this particular amplification curve and plate well with an asterisk in Figure 3B to assist in the visualisation of the correlation between the two analyses and cite this in the figure legend.*
5. Figure 4 C: I do not understand the reason behind treating already purified SARS-CoV-2 RNA with Triton X. This only makes sense in an extraction-free setting.
*The reviewer is correct that the rationale in using Triton-X treated RNA would be applicable for an extraction-free setting. We have included new data in Supplementary Figure 6 that explains the reasoning for having performed the experiment depicted in Figure 4C.*
6. Figure 4 D: When determining the RT-LAMP assay precision, why extracting the RNA 5 times independently and then running the assay? I expect higher variations from the RNA extraction than from the RT-LAMP assay itself, which should be highly stable and reproducible on purified samples unless the sample contains only very few amounts of target RNA and the detection can become stochastic.
*One of the criteria that needed to be met for approving the assay for clinical diagnostics (in collaboration with the accredited clinical diagnostic laboratory and hospital partners) was to insure that for any one sample going through the entire pipeline (including RNA extraction from the original swab sample to final read-out), the result would need to be highly reproducible / precise. Therefore, we performed the exact same assessment our institute used to validate their use of a CE marked RT-qPCR assay, which was to process a nasopharyngeal sample that had an accompanying clinical diagnosis for COVID-19, 5 times from start to finish (lysate to result).*
